# The Management of the Normal Puerperium

**Published:** 1929

**Authors:** H. J. Drew Smythe

**Affiliations:** Assistant Physician-Accoucheur and Gynœcologist, Bristol General Hospital; Gynœcologist, Cossham Memorial Hospital, etc


### THE MANAGEMENT OF THE NORMAL PUERPERIUM.

BY

H. J. Drew Smythe, M.M., M.B., M.S. (Lond.), F.R.C.S. (Eng.),

*Assistant Physician-Accoucheur and Gynæcologist, Bristol General Hospital; Gynæcologist, Cossham Memorial Hospital, etc.*

---

In civilized communities labour cannot be considered a normal physiological function, however much we try to approximate it to this ideal. The civilized woman by her very civilization is rendered abnormal for the purpose of child bearing. She cannot, for example, bear the pains of labour, not because she is hysterical or a coward, but because her senses have been rendered hypersensitive by education and an artificial mode of life. How easy parturition is in a woman of feeble mind, and how tolerant she is of the pains of labour!

The puerperium, too, is not a normal physiological process. It was rendered abnormal when we assumed the upright posture. In the quadruped the pelvic organs are not supported wholly by means of the pelvic floor, and there is no danger of prolapse or malposition of the uterus if the four-footed female goes hunting immediately after giving birth to her young; but in woman uterine displacements are bound to occur, and do occur, if she assumes the upright position immediately or too soon after her labour. The "rational" puerperium is rational for animals and savages but not for the civilized woman, and this is the patient whose puerperium we have to supervise.

The management of labour is more especially concerned with the immediate safety of the mother and child rather than with the future health of the mother. It is during the puerperium that treatment is directed towards the prevention of future morbidity. How often one hears the statement made by patients that they have never been well since the birth of one particular child, and more often than not this was a perfectly normal labour as far as the birth of the child was misconcerned. In a large number of cases this "ill-health" is due, not to the actual labour, but to management of the puerperium. After the child is born the practitioner is apt to leave the management of the patient to the nurse, being only concerned with any deviation from the normal, rather than with the routine treatment of the puerperium.

No rules of treatment can be laid down for every case; each patient has her own particular need, and circumstances will arise when the following routine is impossible, but I think that the management of the puerperium as described will give the most satisfactory results in the majority of cases.

Consider the condition of the bony and muscular structures of the abdomen and pelvis after labour. The pelvis is increased in size by softening and stretching of the ligaments binding the bony pelvis together, and the hips are splayed out, that is, the anterior superior iliac spines are wider apart after than before labour. The muscles of the pelvic floor, especially the pubo— and ischiococcygeus of opposite sides, are stretched and pushed apart by the passage of the child through the genital canal, and there is an area of the pelvic floor containing the vagina which therefore has no muscular support. The fascial coverings of the muscles of the pelvic floor are also distorted and stretched. The uterus is an enlarged heavy organ with all its ligamentous supports, especially the round ligament, softened and stretched by pregnancy. The abdominal wall is stretched, even to the skin, and there is divarication of the recti. If by mischance or mismanagement we have added to these a torn perineum, then we have the loss of the support of the perineum and superficial perineal muscles.

It is at the restoration of these structures to the normal that we should aim in the management of the puerperium. If there has been a tear of the perineum, however slight, its suture is our first duty. Perineal tears should be sutured as soon after the delivery of the placenta and membranes as possible. In extensive tears an anæsthetic is necessary, to allow for accurate approximation of skin, muscle and mucous membrane, as only in this way can one get the absolute quiescence on the part of the patient which is so necessary for a successful repair.

If the patient has had an anæsthetic for the delivery of the child, this may be extended for a while and the suture or sutures for a slight tear put in, but not tied, before the placenta is delivered. For anything beyond a small tear repair should be performed at the end of the third stage, and it is most important that the repair should be thorough.

The mucous membrane of the posterior wall of the vagina should be sutured with catgut separately from the fibro-muscular tissue and skin, which is best sutured with deep silkworm gut sutures, taking a good " bite " of each wall of the tear. The skin may be approximated accurately, after cutting off any bruised edge or tag, by superficial silkworm gut sutures taking up the skin only. In the event of a complete perineal tear, involving the sphincter ani, great care must be taken to approximate the two ends of the sphincter, and catgut is the best suture material. Silk and thread should not be used, as they are not absorbed and form a foreign body which, in the near neighbourhood of the anal canal, is almost certain to become infected, and as a result the whole perineum may break down. Suture of the sphincter is followed by careful suture of the anal mucous membrane, and after that the rest of the perineum is repaired as before described.

The ends of the silkworm gut sutures should not be cut short, as they tend to stick into the patient and cause discomfort. A convenient way of disposing of these ends is to knot the two together about three inches from the original reef knot and cut off the ends immediately distal to this second knot. This leaves a three-inch loop, which can be placed by itself or with others to one side of the tear and wrapped in sterile cotton wool. The advantage of this loop is again manifest when the sutures have to be removed, as a finger can be placed in the loop and the suture retracted. The place for cutting the suture at the edge is thus brought into view, even if the sutures have cut into the skin, as they are inclined to do; and having cut the suture, its extraction is rendered easy by pulling on the loop. It is advisable to tie the patient's legs together until she comes round completely from the anæsthetic, after which it is sufficient to warn her against stretching the thighs apart.

The after treatment of the repair is important; the great essential, as in all wounds, is to keep it dry. This is impossible in puerperal cases, on account of the lochial discharge, but one can keep it as dry as possible by frequent change of pads and by applying a dusting powder of zinc oxide, borax and starch to the perineum. The great vogue and success of iodoform powder as a dressing for these cases was due, not to its antiseptic properties, which are practically negligible, but to its action in keeping the perineum dry. Its obnoxious smell, however, prevents its general use except for cases in which the lochia are offensive, when the iodoform acts as a deodorant.

After micturition or defæcation the perineum should be swabbed clean and the wound touched up with methylated spirits or tr. iodi, and powdered when dry.

The silkworm gut sutures should be removed on the seventh or eighth day, as by then union should have taken place. It is useless to leave them in longer, as by the eighth day after insertion they will have lost their grip of the tissues by cutting in—this can be easily seen by pulling on the loop—and are only acting as tracks for the ingress of bacteria. Immediately after the delivery of the placenta and membranes the patient is given a drachm of liquid extract of ergot by mouth or a tablet of "Femergin," which contains 1/65 gr. of ergotamine tartrate.

If the patient has had an anæsthetic I give a hypodermic injection of 1/130 gr. of ergotamine tartrate at this stage. In my opinion ergotamine tartrate is preferable to pituitary extract for producing uterine contraction; it acts with almost the same rapidity and does not at the same time cause a vaso-constriction. I have seen several cases of extreme collapse taking place within an hour or two hours after the administration of pituitary extract, and the cause of this I am certain is the sudden fall of blood pressure when the effect of the pituitary extract ceases; vaso-dilatation takes the place of constriction, and the patient bleeds into her own capillaries.

We now come to the debatable question of the abdominal binder. Many obstetricians regard this as a useless fetish handed down to us by the midwives and man midwives of the past, but in my opinion it is still a most essential adjunct in the management of the puerperium. To serve any useful purpose it must be applied tightly, and kept tight, and commence well down the thighs in line with the pubes. An extremely efficient type of binder is one which has a series of straps and buckles down the front, by which means the binder can be moulded to the contour of the abdomen and hips and kept tight. Binders secured by safety-pins soon become loose and require frequent adjustment. The object of the binder is twofold. The first, and by far the most important, is to prevent the splaying out of the pelvis which occurs as the result of the softening of the pelvic ligaments and slight widening of the pelvic joints. The binder acts by pulling the pelvis together, and so allowing for the retraction of the ligaments and the return to the normal shape of the pelvis, thus restoring the patient's figure, for which one will receive as many thanks as for the most skilfully conducted labour. The second object is the maintenance of intra-abdominal pressure, the binder taking the place of the slack abdominal wall. The maintenance of the intra-abdominal pressure is said to help the contraction of the uterus and so prevent post-partum hemorrhage, and also aid in the involution of the uterus. These results are, I think, extremely doubtful, and are more than counterbalanced by the binder preventing the proper working and use of the abdominal muscles. Were this, namely the maintenance of intra-abdominal pressure, the only object of the binder, I would discontinue its use ; but its aid in the return of the pelvis to normal is of great value. It is also a great comfort to the patient herself in preventing that feeling of the pelvis falling apart of which many complain who have not had a binder applied. Lastly, it serves as a point of attachment for the pads.

The position of the patient in bed is a most important feature of the management. No longer does one keep the patient lying flat on her back unless there is a definite reason, as after post-partum hemorrhage, and even then only as long as there is danger of cerebral anæmia. As soon as the patient has been made comfortable after delivery, she is put in the Fowler position or with high " head " blocks, and a " donkey " placed under the knees. This position can be improvised in a private house by placing the head of the bed on the seats of two kitchen chairs. The advantage of this position is that the lochia can completely drain away from the vagina, whereas in the dorsal recumbent position the discharge collects in the vagina and simply overflows on to the pad, and this collection of lochia is a direct invitation to invasion by micro-organisms. Also, should the uterus become infected and the infection spread to the peritoneum, it will, in the majority of cases, be limited to the pelvis, if the patient is in the Fowler position. The patient should be told that she may turn on her side to sleep if she wishes ; in fact, there is no contra-indication in a normal healthy patient, except the presence of stitches, which will prevent her from assuming this position at any time. Even with stitches present, provided the thighs are tied together, she can lie on her side.

During the first day of the puerperium attention should be paid to the bladder and breasts. The bladder should be emptied regularly and the urine not allowed to accumulate to the point of distention, as a distended bladder prevents contraction of the uterus, and invites urinary infection. The patient should be encouraged to empty the bladder naturally and only as a last resource should a catheter be used, as some patients get into a "catheter habit" which may last for several days. On the other hand, the bladder should not be allowed to become distended, as this may lead to the patient being unable to pass urine at all owing to atony of the bladder, or she will only pass a small quantity at each act of micturition; under these circumstances catheterization is essential. In this class of case hexamine gr. 10 and acid sodium phosphate gr. 30 given three times a day will prevent urinary infection and produce a certain amount of vesical irritability, which will give rise to the desire for natural micturition at fairly frequent intervals, so preventing over-distention and the need for catheterization.

The breasts should have already been attended to during pregnancy and any defects in the nipples treated. This treatment should be continued during the puerperium. The nipples should be hardened with Eau de Cologne or other spirit and, if indrawn, manipulated by the doctor's or nurse's fingers, aided if necessary by the use of the breast pump. If the nipple projects sufficiently to be grasped by the child's mouth, then the normal projection will soon be produced by the child's suckling. The child should be put to the breast regularly from the very first day, as this stimulates the breast glands to secrete and the uterus to contract, and accustoms the child to take the breast. The hours of feeding should be regular; whether three-hourly or four-hourly, and whether at night or not, depending entirely on the size and nutrition of the child. No definite rule can be laid down for every child, but in normal circumstances four-hourly feeds are best with an eight-hour interval from 10 to 6 at night between feeds ; this last gives the mother a sufficient night's rest which is so essential, and it also rests the breasts. Strict attention must be paid to the cleansing of the nipples before and after feeds, and any abrasion noted and treated.

On the night of the second day an aperient must be given, and castor oil is best for this purpose, as the desired result is sure and there is no effect on the child. After the second day the bowels can be regulated, if necessary, by means of liquid paraffin and cascara evacuant either once or twice a day, or by one of the liquid paraffin preparations in combination with agar-agar.

The mother's diet should be plain and nourishing, avoiding anything which may indirectly affect the child's digestion, otherwise she may have a normal diet after the bowels have been opened on the morning of the third day. Between meals she should have plenty of milk to drink, at least two pints of milk during the day, either as plain milk or cocoa made with milk or made up in the form of groats, which is an excellent galactogogue. Strong tea and coffee should be avoided and alcohol is unnecessary. The value of stout as a galactogogue is questionable, and in the majority of cases it produces adipose tissue rather than breast milk.

During the fourth and fifth days the breasts may become engorged with milk, and care must be taken to see that this engorgement is relieved, either by the child or by means of massage of the breasts and attention to the bowels. The child may be frightened from taking the breast during this engorgement, or at any time that the breast milk is excessive, on account of the sudden stream of milk which is projected into his mouth and chokes him when he first seizes the nipple. This may be prevented by drawing off a small quantity of milk by means of the breast pump before putting the child to the breast.

On the fifth day of the puerperium abdominal exercises must be commenced, and I regard these as one of the most important items in the prevention of future morbidity.

Before commencing the exercises the binder is undone in its upper abdominal half, so as to allow free play for the abdominal muscles. The first exercise consists in raising the head and shoulders from the bed while the patient is lying flat on her back with the arms by the sides. This is done twice a day, and regulated by the nurse so that it does not unduly fatigue the patient. Exercises should never be carried out to the point of fatigue. At first this exercise can only be done for a few minutes, but after a day or so longer time can be devoted to exercises and fresh ones introduced. The object of this particular exercise is to strengthen the recti and indirectly all the abdominal muscles.

After the exercises the patient turns over and lies flat on the abdomen, which may be supported at first by means of a pillow placed under it, but this should be discontinued later. This position throws the uterus forward and so rests the strain on the round ligaments, and if this is done for an hour or longer twice a day the chances of the uterus becoming retroverted are extremely small. Retroversion is in the majority of cases due to the patient lying on her back during the whole of the puerperium. The constant strain on the round ligaments produced by the action of gravity on the heavy, involuting uterus is the main cause, and this is inevitable while the patient is in the supine position. Most patients get used in a short time to lying on the abdomen, and will read their books, which are propped up against the pillow, and many times I have found them, having fallen asleep while reading, in this position.

After the seventh day the patient is no longer kept in the Fowler position at night, but allowed to sleep as she would normally, and encouraged to sleep lying on her side rather than on her back.

Exercises are continued as before and fresh ones added. In the first of these the patient, lying on her back with arms to the sides and binder removed, slowly raises in turn first one and then the other lower limb with the knee held rigid. Later both legs are raised together, and then the lower half of the trunk, the weight being taken by the shoulders and the arms, which are held stiffly on the bed. In another exercise, with the patient lying on her side, the leg stroke in swimming is imitated, and this is repeated on turning over to the opposite side.

By means of these exercises the muscles of the abdomen and pelvis are brought back to their normal tone, and all laxness is taken up before they have to bear the weight of the abdominal and pelvic contents, with the result that after-complications of pregnancy, such as visceroptosis and prolapse, are to a considerable extent prevented.

These exercises can be performed under the supervision of the midwife, and do not require the services of a masseuse; in fact, they can be performed without supervision if the warning against the production of fatigue is given and heeded. Thus there is no extra drain on the patient's financial resources, and as a consequence exercises can be performed by the poorest as well as the richest of our patients. If, however, the patient can afford a masseuse they can be performed under her direction, and their benefit greatly enhanced by the addition of abdominal massage. Massage not only increases the muscular tone but disperses the superfluous abdominal fat, which tends to collect during pregnancy and the puerperium, and increases the elasticity of the skin.

The patient is allowed to sit out of bed daily, from the tenth to the fourteenth day, and the abdominal binder may now be discarded. It is better to keep a mother in bed for fourteen days, but circumstances, financial and otherwise, may not permit of this. In every case, before she is allowed up, it is a *sine qua non* that the uterus can no longer be felt on abdominal palpation and that the lochia are clear and normal.

Abdominal exercises should be continued even when the patient is sitting out of bed each day, and if this is done it will be found that she will be able to walk quite steadily at her first attempt, and there is very little, if any, giddiness or stooping such as one sees in other patients who have not had exercises.

Involution takes from six to eight weeks to complete, and it is necessary to warn the patient before leaving our care that she must not lift any heavy weight, especially in a stooping position, for at least two months after her baby's birth.

So we bring the mother to the time, either three weeks or a month after parturition, depending on circumstances chiefly financial, when she must resume again her daily duties. It is our hope that, by strict attention to the management of the puerperium, the mother is, and will be in the future, a perfectly healthy woman.

